# Repression of Human T-lymphotropic virus type 1 Long Terminal Repeat sense transcription by Sp1 recruitment to novel Sp1 binding sites

**DOI:** 10.1038/srep43221

**Published:** 2017-03-03

**Authors:** Sylvain Fauquenoy, Gwenaëlle Robette, Anna Kula, Caroline Vanhulle, Sophie Bouchat, Nadège Delacourt, Anthony Rodari, Céline Marban, Christian Schwartz, Arsène Burny, Olivier Rohr, Benoit Van Driessche, Carine Van Lint

**Affiliations:** 1 Service of Molecular Virology, Department of Molecular Biology (DBM), Université Libre de Bruxelles (ULB), Rue des Professeurs Jeener et Brachet 12, 6041 Gosselies, Belgium; 2 Biomaterials and Bioengineering, Inserm UMR 1121, Faculty of Dentistry, University of Strasbourg, France; 3 Institut Universitaire de Technologie Louis Pasteur, University of Strasbourg, Schiltigheim, France; 4 Laboratory of Dynamic of Host-Pathogen Interactions (DHPI), EA7292, University of Strasbourg, Strasbourg, France; 5 Laboratory of Experimental Hematology, Institut Jules Bordet, Université Libre de Bruxelles (ULB), Brussels, Belgium

## Abstract

Human T-lymphotropic Virus type 1 (HTLV-1) infection is characterized by viral latency in the majority of infected cells and by the absence of viremia. These features are thought to be due to the repression of viral sense transcription *in vivo*. Here, our *in silico* analysis of the HTLV-1 Long Terminal Repeat (LTR) promoter nucleotide sequence revealed, in addition to the four Sp1 binding sites previously identified, the presence of two additional potential Sp1 sites within the R region. We demonstrated that the Sp1 and Sp3 transcription factors bound *in vitro* to these two sites and compared the binding affinity for Sp1 of all six different HTLV-1 Sp1 sites. By chromatin immunoprecipitation experiments, we showed Sp1 recruitment *in vivo* to the newly identified Sp1 sites. We demonstrated in the nucleosomal context of an episomal reporter vector that the Sp1 sites interfered with both the sense and antisense LTR promoter activities. Interestingly, the Sp1 sites exhibited together a repressor effect on the LTR sense transcriptional activity but had no effect on the LTR antisense activity. Thus, our results demonstrate the presence of two new functional Sp1 binding sites in the HTLV-1 LTR, which act as negative *cis*-regulatory elements of sense viral transcription.

The complex retrovirus HTLV-1 (Human T-lymphotropic virus type 1) infects about 15–20 million people worldwide and is endemic in Japan, Africa, South America and the Caribbean islands^1^,^2^. The virus is the etiologic agent of an aggressive and rapidly fatal leukemia called ATLL (Adult T Cell Leukemia/Lymphoma)^3^,^4^ and an inflammatory disease named TSP/HAM (Tropical Spastic Paraparesis/HTLV-1 Associated myelopathy)^5^. While the majority of infected people remain lifelong clinically asymptomatic carriers, a small proportion of them develop one of the diseases after a long period of latency^6^,^7^. The infection is characterized by the absence of viremia due to virus latency in most infected cells and this is explained by a repression of viral sense transcription *in vivo*^8^,^9^ from the 5′-LTR. Latency represents a viral strategy to escape from the host immune system and likely allows for tumor progression^10^.

Transcription of HTLV-1 genes (except *hbz*), integrated into host chromosomal DNA after reverse transcription, initiates at the U3/R junction in the 5′-LTR and is regulated by cellular factors binding to the 5′-LTR, by the viral transactivating Tax protein and by the chromatin organization of the HTLV-1 provirus^11^. The 5′-LTR contains several cellular transcription factor binding sites (Fig. 1), including the TRE-1 (Tax Responsive Element 1) which carries three imperfectly conserved 21-bp enhancer elements, named viral cAMP-response elements (CREs) (reviewed in ref. 12) and binds CREB/ATF (cAMP-responsive element-binding protein/activating transcription factor) family members, and the TRE-2 which has been demonstrated to collaborate with the promoter proximal TRE-1 via recruitment of Ets, Elk-1 and Sp1^13^,^14^,^15^. Both the TRE-1 and TRE-2 motifs are required in *cis* to allow Tax transactivation of the HTLV-1 promoter^11^. Moreover, the complementary strand of the proviral genome X region encodes the HTLV-1 basic leucine zipper factor (HBZ) which is regulated by an independent antisense transcriptional promoter located in the 3′-LTR^16^.

Sp (Specificity protein) factors are member of the zing-finger Specificity protein/Krüppel-like Factor (SP/KLF) transcription factor family which is characterized by a highly conserved DNA-binding domain in ref. 17. To date, four members have been identified (Sp1, Sp2, Sp3 and Sp4)^18^,^19^. Sp1, Sp3 and Sp4 bind to GC-boxes, while Sp2 binds to GT-boxes. Sp1 and Sp3 are ubiquitously expressed^20^, while Sp4 expression is restricted to neuronal cells^21^. Sp1 binds to more than 1000 different cellular promoters^22^ and plays a role in transcriptional activation, repression and maintenance of basal transcription of cellular genes involved in cell proliferation, apoptosis and differentiation, and of viral genes^23^. Sp1 was initially identified as a constitutive transcription activator of housekeeping genes and TATA-less genes which are not highly regulated^17^, but Sp1 has been shown either to enhance or to repress transcriptional activity of many genes involved in a wide range of biological processes^24^. Sp1 can interact and recruits a large number of proteins including histone-modifying enzymes (such as p300, CBP (CREB-binding protein), HDAC1 (Histone Deacetylase) and HDAC2) and chromatin remodelling complex (such as SWI/SNF) to activate or repress gene expression. Sp1 plays a dual role in the regulation of gene expression (reviewed in ref. 17). For example in the case of the CDKN1A/p21 (cellular cyclin-dependent kinase inhibitor) gene promoter which harbours six Sp1 binding sites, Sp1 cooperates with the transcriptional coactivators p300 and CBP (CREB-binding protein) which possess histone acetyltransferase (HAT) activity to transactivate the cellular p21 promoter^25^. Inversely, our laboratory has previously demonstrated that Sp1 recruits the cellular cofactor CTIP2/Bcl11b (COUP-TF Interacting Protein 2/B cell leukemia/lymphoma 11b) to the proximal region of the cellular p21 promoter and to the viral HIV-1 (Human Immunodeficiency Virus type 1) LTR promoter and represses gene expression in cooperation with HDACs and histone methyltransferases (HMTs)^26^,^27^,^28^. Sp3 transcription factor has also been demonstrated as an activator or a repressor of gene expression due to the differential acetylation status of the protein^29^. Sp1 and Sp3 share exactly the same consensus binding sites, but their binding properties to DNA and regulatory functions are different, depending on the promoter context and cellular background^30^,^31^,^32^.

Several Sp1 binding sites have been previously identified in the HTLV-1 LTRs^33^,^34^,^35^,^36^ and are presented in Fig. 1a and b. Two Sp1 binding sites located in the U3 5′-LTR region (one between CRE-2 and CRE-3, referred to as Sp1#1 and the other in CRE-3, referred to as Sp1#2) are critical for basal and Tax-transactivated promoter expression^33^,^37^,^38^, while the Sp1 binding site located in the U5RE enhancer (referred to as Sp1#5) acts as a repressor of HTLV-1 gene expression in the absence of Tax^35^. The Sp1 binding sites located within the U5 region of the 3′-LTR (referred to as Sp1#5 and Sp1#6) are critical for transcription of the *hbz* gene, which is transcribed as an HTLV-1 antisense transcript from the 3′-LTR^16^,^39^.

However, these studies have been performed in various cell lines which are not all the main target of HTLV-1 (i.e. CD4+T cells). Moreover, none of these previous studies have been performed with a complete HTLV-1 LTR, thereby excluding the potential functional synergy or competition between the different Sp1 binding sites and other *cis*-regulatory elements present in the HTLV-1 LTR.

Since Sp1 is an important regulator of eukaryotic promoter transcriptional activity, we here analyzed *in silico* the HTLV-1 LTR nucleotide sequence for the presence of other potential Sp1 binding sites in addition of the four Sp1 sites previously described. Interestingly, we identified two new Sp1 binding sites in the R region of the HTLV-1 LTR (Fig. 1, referred to as Sp1#3 and Sp1#4) and we characterized them physically and functionally. Importantly, we studied for the first time the role of all six viral Sp1 sites in the nucleosomal context of an episomal reporter vector and the contribution of each Sp1 site to both the sense and antisense transcriptional activities of the HTLV-1 LTR in a CD4^+^ T-cell line.

## Results

### Sp1 transcription factor specifically interacts with each of the two newly identified Sp1 binding sites located in the R region of the HLTV-1 promoter

**Figure 1:**
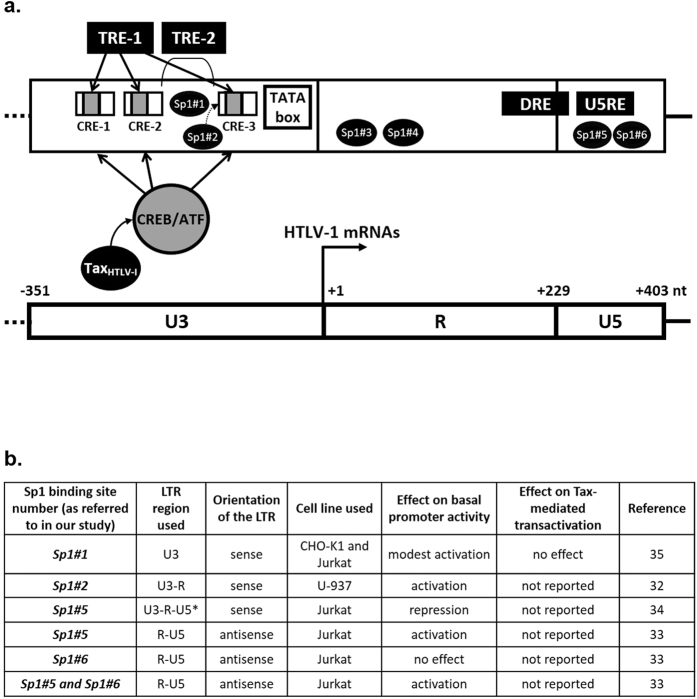
Transcription factor-binding sites in the 5′-LTR of the HTLV-1 genome. (**a**) The 5′-LTR contains a TATA box (nt −30/−21) upstream of the transcription initiation site at the U3-R junction (mRNA start site at + 1 is indicated by an *arrow*). The TRE-1 (Tax responsive element 1) is three imperfectly repeated sequences of 21-bp named viral cAMP-response elements (CREs; referred to as CRE-1 nt-253/−233, CRE-2 nt-203/−183 and CRE-3 nt −104/−84). These sequences are major transcriptional enhancers, which interact with the cellular transcription family factors CREB/ATF. Transcriptional activation of the HTLV-1 LTR by the viral encoded Tax_HTLV-1_ transactivator requires these enhancers. The U3 region also contains the TRE-2 (Tax responsive element 2, nt −163/−117) which is critical for Tax-mediated transactivation of the HTLV-1 LTR and interact with cellular transcription factors Ets and Sp1. Outside of U3, the DRE (Downstream regulatory element, nt + 195/−240) in the R region, interacting with YB-1 and critical for basal gene expression, and the U5RE (U5 repressive element) in the U5 region are regulatory sequences important for HTLV-1 gene expression. There are several Sp1 binding sites in the HTLV-1 LTR. Sp1 binds to the U3 region of HTLV-1 LTR at two elements, referred to as Sp1#1 (nt −142/−132) and Sp1#2 (nt −105/−91), and to the U5 region, referred to as Sp1#5 (nt + 269/−282) and Sp1#6 (nt + 345/ + 368). We identified and characterized the two Sp1 binding sites located in the R region, referred to as Sp1#3 (nt + 20/ + 33) and Sp1#4 (nt + 72/ + 85). (**b**) The effects of the different Sp1 binding sites on HTLV-1 transcription as established before the present study are summarized.

**Figure 2:**
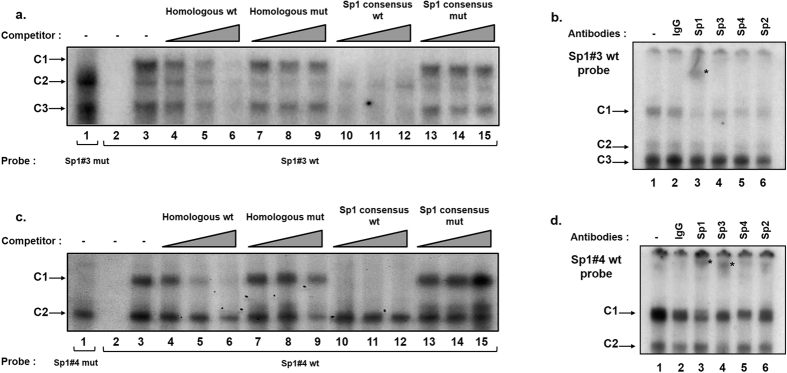
Characterization of nuclear factors interacting with the Sp1#3 and Sp1#4 putative binding sites located in the R region of the HLTV-1 promoter. The Sp1#3 mut (lane 1) and Sp1#3 wt (lanes 2 to 15) probes (**a**) or the Sp1#4 mut (lane 1) and Sp1#4 wt (lane 2 to 15) probes (**c**) were incubated alone (lane 2) or with nuclear extracts (10 μg) from Jurkat T cells (Jurkat NE) in the absence (lanes 1 and 3) or in the presence of increasing concentrations (4-, 16- and 64-fold molar excesses) of the homologous Sp1#3 oligonucleotide (lanes 4 to 6), of the mutated homologous Sp1#3 mut oligonucleotide (lanes 7 to 9) (**a**), or the homologous Sp1#4 oligonucleotide (lanes 4 to 6) of the mutated homologous Sp1#4 mut oligonucleotide (lanes 7 to 9) (**c**), of the Sp1 binding site consensus oligonucleotide (lanes 10 to 12) or of the mutated Sp1 binding site consensus oligonucleotide (lanes 13 to 15) **(a** and **c**). The figure shows the specific retarded bands of interest, which are indicated by arrows. The terms C1, C2, and C3 refer to complexes 1, 2 and 3 in (**a**) and the terms C1 and C2 refer to complexes 1 and 2 in (**c**). Nuclear extracts (10 μg) from Jurkat T cells were incubated, before the addition of the Sp1#3 wt probe (**b**), or the Sp1#4 wt probe (**d**), either with a purified rabbit IgG as a negative control (lane 2), or with an antibody directed against Sp family members including Sp1 (lane 3), Sp2 (lane 6), Sp3 (lane 4), Sp4 (lane 5) or without antibody (lane 1). The figure shows the specific bands of interest indicated by arrows. Supershifted complexes are indicated by asterisks.

*In silico* analyses of the HTLV-1 LTR sequence revealed the presence of two new putative Sp1 binding sites located in the R region of the HTLV-1 promoter (designated site Sp1#3 from nt +23 to +37 and designated site Sp1#4 from nt +72 to +84, respectively, where nt +1 is the transcription start site, Fig. 1a). In order to identify cellular factors that bind to these potential Sp1 motifs, we characterized physically these two new sites by performing EMSAs and supershift EMSAs using as probes a 33-bp Sp1#3-containing oligonucleotide corresponding to positions nt +17 to +50 of the LTR and a 35-bp Sp1#4-containing oligonucleotide corresponding to positions nt +64 to +99 of the LTR (Supplementary Table S1). The radiolabeled double-stranded probe Sp1#3 was incubated with nuclear extracts from Jurkat T cells and three major protein-DNA retarded complexes were observed and designed Sp1#3 C1 to Sp1#3 C3 (Fig. 2a, lane 3). To evaluate the specificity of these interactions, unlabeled homologous oligonucleotides were prepared and used as competitors in the EMSAs. The formation of the three retarded complexes was competed out by molar excesses of the unlabeled homologous Sp1#3 oligonucleotide (Fig. 2a, lanes 4 to 6), thereby demonstrating the sequence specificity of the complexes binding to the Sp1#3 probe. Furthermore, the addition of similar molar excesses of an oligonucleotide corresponding to an Sp1 binding site consensus (Fig. 2a, lanes 10 to 12) hindered the formation of the Sp1#3 C1 and C3 complexes, whereas formation of these latter complexes was not affected by the addition of the same molar excesses of a mutated version of the Sp1 consensus oligonucleotide (Fig. 2a, lanes 13 to 15), thereby demonstrating that these complexes were specific to the Sp1 motif. To abolish factor binding to the Sp1#3 motif, we designed a mutation (designated Sp1#3 mut) consisting in the substitution of the two central nucleotides CC with the dinucleotide AA and we performed competition EMSAs with the Sp1#3 wild-type oligonucleotide as a probe (Fig. 2a, lanes 7 to 9). The Sp1-specific retarded complexes C1 and C3 were not competed out by addition of molar excesses of the Sp1#3 mut unlabeled oligonucleotide. Moreover, complex C1 was absent when we incubated nuclear extracts from Jurkat T cells with the radiolabeled probe Sp1#3 mut (Fig. 2a, lane 1), demonstrating that selected mutations abolished the binding of proteins associated to the Sp1#3 binding site in C1 complex.

To identify the factors present in the retarded complexes, we performed supershift assays using antibodies directed against individual members of the transcription factors Sp family (Fig. 2b). Radiolabeled probe Sp1#3 was incubated with nuclear extracts from Jurkat T cells and either a purified rabbit IgG (Fig. 2b, lane 2) as a negative control or an antibody directed against Sp1, Sp3, Sp4 or Sp2 (Fig. 2b, lanes 3 to 6). The addition of an antibody directed against Sp1 interfered with the formation of complex Sp1#3 C1 and led to the appearance of a supershifted complex of decreased mobility (Fig. 2b, lane 3). Moreover, no retarded band was affected by the addition of a purified rabbit IgG (Fig. 2b, lane 2), indicating the specificity of the protein-antibody interactions.

Incubation of the radiolabeled Sp1#4 probe with nuclear extracts from Jurkat T cells resulted in the formation of two retarded complexes designated Sp1#4 C1 and Sp1#4 C2 (Fig. 2c, lane 3). The formation of the two retarded complexes was competed out by molar excesses of the unlabeled homologous Sp1#4 oligonucleotide (Fig. 2c, lanes 4 to 6), thereby demonstrating the sequence specificity of the complexes binding to the Sp1#4 probe. Molar excesses of the unlabeled Sp1 consensus oligonucleotide (Fig. 2c, lanes 10 to 12), but not of its mutated version (Fig. 2c, lanes 13 to 15), specifically inhibited the formation of the C1 complex, whereas no difference in C2 complex formation was observed, thereby demonstrating that the Sp1#4 C1 complex was specific to the Sp1 motif. To abolish factor binding to the Sp1#4 motif, we designed a mutation (designated Sp1#4 mut) and performed competition EMSAs with the Sp1#4 wild-type oligonucleotide as a probe (Fig. 2c, lanes 7 to 9). The Sp1-specific retarded complex C1 was not competed out by addition of molar excesses of the Sp1#4 mut unlabeled oligonucleotide. Moreover, the C1 complex was absent when we incubated nuclear extracts from Jurkat T cells with the radiolabeled probe Sp1#4 mut (Fig. 2c, lane 1), demonstrating that selected mutations abolish the binding of proteins associated to the Sp1#4 binding site in complex C1. Supershift assays showed that addition of an antibody directed against Sp1 interfered with the formation of complex Sp1#4 C1, and that addition of an antibody directed against Sp3 weakly decreased the intensity of the complex Sp1#4 C1, leading to the appearance of a supershifted complex of decreased mobility with both antibodies (Fig. 2d, lane 3 and lane 4, respectively).

Together, our results identify *in vitro* two new functional Sp1 binding sites in the HTLV-1 R region (Sp1#3 and Sp1#4). We demonstrate that the Sp1 transcription factor interacts *in vitro* with these two new Sp1 binding sites and that the Sp3 transcription factor interacts only with the Sp1#4 binding site.

### Ranking of the HTLV-1 Sp1 binding sites with respect to their affinity for Sp1

**Figure 3:**
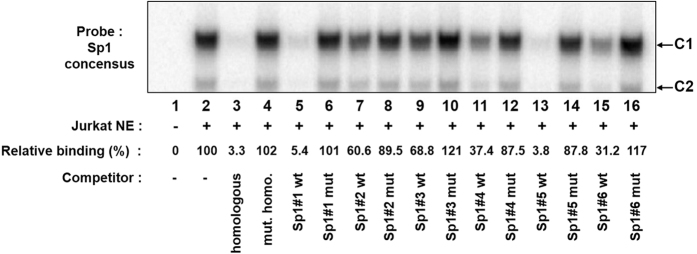
Ranking of the LTR Sp1-binding sites with respect to their affinity for Sp1. The Sp1 binding site consensus (lanes 1 to 16) probe was incubated alone (lane 1) or with nuclear extracts (10 μg) from Jurkat T cells in the absence of competitor (lane 2) or in the presence of a molar excess (30 fold) of a competitor corresponding to the homologous consensus Sp1 binding site (lane 3), to the mutated consensus Sp1 binding site (lane 4), or to the indicated wild-type (lanes 5, 7, 9, 11, 13, 15) or mutated (lanes 6, 8, 10, 12, 14, 16) HTLV-1 Sp1 binding site. The figure shows the specific retarded bands of interest, which are indicated by arrows. The terms C1 and C2 refer to complexes 1 and 2. Quantification of EMSAs was performed with a PhosphorImager and is shown for the C1 complex. The results are expressed as a percentage of binding in comparison to binding in the absence of competitor oligonucleotide.

In order to compare the affinity for Sp1 of the six Sp1 binding sites located in the HTLV-1 LTR (see Fig. 1a), we performed competition assays with a probe corresponding to the Sp1 binding site consensus. This radiolabeled probe was incubated with nuclear extracts from Jurkat T cells in the absence (Fig. 3, lane 2) or in the presence of the same molar excess of different unlabelled oligonucleotides as competitors, corresponding either to the homologous Sp1 consensus oligonucleotide (Fig. 3, lane 3), to the mutated homologous sequence (Fig. 3, lane 4), to the six wild-type Sp1 binding sites of the HTLV-1 promoter (Fig. 3, lanes 5, 7, 9, 11, 13, and 15, respectively) and to their mutated versions (Fig. 3, lanes 6, 8, 10, 12, 14, and 16, respectively). Incubation of the Sp1 consensus probe with nuclear extracts from Jurkat T cells resulted in the formation of two broad retarded complexes (Fig. 3, lane 2), which were competed out by the unlabeled homologous Sp1 consensus oligonucleotide (Fig. 3, lane 3), but not by the mutated Sp1 consensus oligonucleotide (Fig. 3, lane 4), demonstrating the sequence specificity of the two complexes binding to the Sp1 consensus motif. Interestingly, the addition of an unlabeled oligonucleotide corresponding to the Sp1#1 binding site hindered the formation of the two complexes (Fig. 3, lane 5). The addition of an unlabeled oligonucleotide corresponding to the mutated Sp1#1 binding site (designed Sp1#1 mut) did not affect retarded complex formation (Fig. 3, lane 6), indicating that the HTLV-1 Sp1#1 site is a high affinity Sp1 binding site and confirming that its mutation abolishes Sp1 binding to this Sp1#1 site. We obtained similar results with the Sp1#5 binding site (Fig. 3, lanes 13 and 14). Moreover, formation of two retarded complexes was only slightly decreased by the addition an unlabelled oligonucleotide corresponding to the Sp1#2, Sp1#3, Sp1#4 and Sp1#6 binding sites (Fig. 3, lanes 7, 9, 11, and 15, respectively), suggesting a higher affinity for Sp1 of the Sp1 consensus motif than that of the HTLV-1 Sp1#2, Sp1#3, Sp1#4 and Sp1#6 binding sites. The mutated versions of the LTR Sp1 binding sites did not affect the two retarded complexes observed with the Sp1 consensus motif (Fig. 3, lanes 6, 8, 10, 12, 14, and 16, respectively), indicating that Sp1 was not able to bind to these mutated Sp1 binding sites.

Phosphorimaging analyses were used to quantify the intensity of the band corresponding to complex C1 (Fig. 3, relative binding). The results were expressed as percentages of binding of Sp1 to the consensus Sp1 site in the absence or presence of the six HTLV-1 wild-type or mutated competitor oligonucleotides. We arbitrarily assigned a value of 100% of binding to the band corresponding to complex C1 observed when the Sp1 consensus probe was incubated with nuclear extracts in the absence of competitor (Fig. 3, lane 2). We observed the strongest decrease in the intensity of the C1 complex with the unlabeled oligonucleotide corresponding to the Sp1 consensus, the Sp1#1 wild-type or the Sp1#5 wild-type competitors (similar percentages of relative binding: 3.3%, 5.4% and 3.8%, respectively). The unlabelled oligonucleotides corresponding to the Sp1#4 wild-type and the Sp1#6 wild-type binding sites moderately decreased the intensity of the C1 complex (37.4% and 31.2% of relative binding, respectively). Finally, the Sp1#2 wild-type and Sp1#3 wild-type binding sites only weakly decreased C1 complex formation (60.6% and 68.8% of relative binding, respectively). These analyses allowed the ranking of the HTLV-1 LTR Sp1 binding sites with respect to their affinity for Sp1: Sp1#5 ≥ Sp1#1 > Sp1#6 > Sp1#4 > Sp1#2 > Sp1#3.

Taken together, our results suggest that the HTLV-1 Sp1#1 and Sp1#5 binding sites have the highest binding affinity for the Sp1 transcription factor, among all the LTR Sp1 binding sites identified so far. The new Sp1#4 binding site ranks in the middle of the range, while the Sp1#3 binding site exhibits the weakest affinity.

### Multimerized copies of the Sp1#3 and Sp1#4 binding sites confer Sp1 inducibility to a heterologous minimal promoter in *Drosophila* cell line

Next, we characterized the functionality of the newly identified HTLV-1 Sp1 binding sites (Sp1#3 and Sp1#4) out of the context of the whole HTLV-1 LTR. To examine whether the Sp1 transcription factor acts through the Sp1#3 and Sp1#4 binding sites, we produced artificial luciferase reporter constructs in which multimerized copies of wild-type and mutated Sp1#3 + 4 binding sites were inserted into the pTK-luc reporter construct immediately upstream of the TK-luciferase transcriptional unit in the sense or antisense orientation. These plasmids were referred to as p (Sp1#3 wt + 4 wt)_3_senseTK-luc and p (Sp1#3 wt + 4 wt)_3_antisenseTK-luc, respectively. The resulting constructs were cotransfected with increasing amounts of an expression vector for Sp1 and the internal control vector pRL-TK (in which the herpes simplex virus thymidine kinase promoter drives *Renilla* luciferase gene expression) in *Drosophila* Schneider SL-2 cells (which unlike most mammalian cells lack endogenous Sp factors). At 24 hours post-transfection, cell lysates were assayed for luciferase activity.

**Figure 4:**
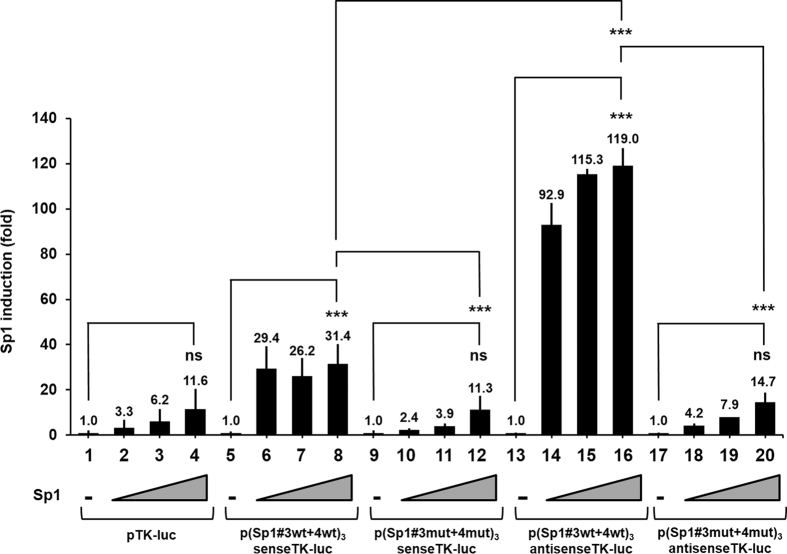
Ability of multimerized HTLV-1 Sp1#3 and Sp1#4 putative binding sites to confer Sp1 stimulation to a TK minimal promoter in *Drosophila* SL-2 cell line. SL-2 cells were cotransfected with 100 ng of a TK luciferase reporter construct devoid (lanes 1 to 4) or not of upstream wild-type or mutated Sp1#3 and Sp1#4 motifs (as indicated, lanes 5 to 20) and increasing amounts (0, 25, 50 and 100 ng) of an Sp1 expression vector (pPacSp1). To maintain the same amount of transfected DNA and to avoid squelching artifacts, the different amounts of pPacSp1 cotransfected were complemented to 100 ng of DNA by using the corresponding empty plasmid. Each transfection included 5 ng of the internal control plasmid, pRL-TK, in which the herpes simplex virus thymidine kinase promoter drives *Renilla* luciferase gene expression. Luciferase activities (Firefly and *Renilla*) were measured in cell lysates 24 h after transfection. The results are expressed as luciferase_firefly_/luciferase_*Renilla*_ and presented as histograms indicating the induction by Sp1 (in fold) with respect to the activity of each TK reporter construct in the absence of Sp1, which was assigned a value of 1. Means and standard errors of the means from triplicate samples are represented. An experiment representative of three independent experiments is shown. ns corresponds to a p value > 0.05, ***Corresponds to a p value < 0.001 in ANOVA test.

As shown in Fig. 4, addition of three copies of the wild-type Sp1#3 + 4 motif in the sense (Fig. 4, lanes 5 to 8) or antisense (Fig. 4, lanes 13 to 16) orientation upstream of the TK promoter resulted in an increase of luciferase activity by ectopically expressed Sp1 (up to 31.4-fold or 119-fold, respectively). These results indicate that Sp1#3 + 4 binding sites exhibit Sp1-dependent stimulatory effect in the *Drosophila* cell line model and that this effect is stronger in the antisense orientation than in the sense orientation. This Sp1 stimulatory effect required intact Sp1#3 + 4 motifs, because mutations in these motifs [p (Sp1#3 mut + 4 mut)_3_senseTK-luc and p (Sp1#3 mut + 4 mut)_3_antisenseTK-luc] resulted in levels of Sp1-mediated transactivation similar to those obtained with the parental control pTK-luc vector (Fig. 4, lanes 9 to 12 and lanes 17 to 20, respectively).

We conclude from these experiments that ectopic Sp1 protein had an HTLV-1 Sp1#3 + 4-dependent stimulatory effect on the heterologous TK promoter containing multiple upstream Sp1#3 + 4 motifs. This stimulatory effect occurred preferentially when the Sp1#3 + 4 motif was cloned in the antisense orientation. These results thus establish, in the context of a minimal luciferase reporter vector containing only the two newly identified HTLV-1 Sp1 binding sites, the functional significance of Sp1 through the viral Sp1#3 + 4 motifs.

### Mutations in the R region Sp1 binding sites increase basal gene expression from the HTLV-1 sense LTR to a greater extent than the individual mutations

**Figure 5:**
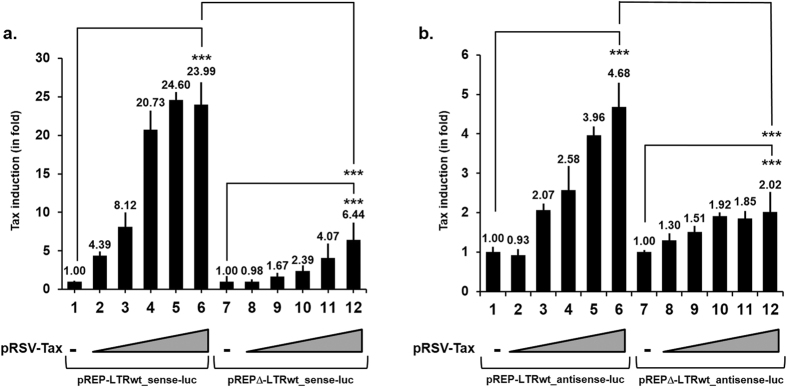
Comparison of the Tax inductibility of the HTLV-1 promoters in non episomal and episomal reporter vectors. Jurkat T cells were transiently cotransfected with 600 ng of either pREP-LTRwt_sense-luc ((**a**) lanes 1 to 6), pREP∆-LTRwt_sense-luc ((**a**) lanes 7 to 12), pREP-LTRwt_antisense-luc ((**b**) lanes 1 to 6) or pREP∆-LTRwt_antisense-luc ((**b**) lanes 7 to 12) and increasing amounts of a HTLV-1 Tax expression vector pRSV-Tax (0, 25, 50, 100, 200 and 400 ng of plasmid DNA). To maintain the same amount of transfected DNA and to avoid squelching artifacts, the different amounts of pRSV-Tax cotransfected were complemented to 400 ng of DNA by using the corresponding empty plasmid. Each transfection included 10 ng of the internal control *Renilla* luciferase plasmid, pRL-TK. Luciferase activities (Firefly and *Renilla*) and protein concentrations were measured in cell lysates 48 h after transfection. The results are expressed as luciferase_firefly_/luciferase_*Renilla*_/[protein] and presented as histograms indicating the induction by Tax (in fold) with respect to the activity of each LTR reporter construct in the absence of Tax, which was arbitrarily assigned a value of 1. Means and standard errors of the means from triplicate samples are presented. An experiment representative of three independent experiments is shown. ns corresponds to a p value > 0.05, ***Corresponds to a p value < 0.001 in ANOVA test.

Since transiently transfected plasmid DNA does not always form proper chromatin structure, we decided to address the function of previously and newly identified HTLV-1 Sp1 binding sites in the context of an episomal reporter vector. So far, the literature has not reported gene expression studies on the HTLV-1 LTR in such a nucleosomal context. To this end, we cloned the complete HTLV-1 LTR (from nt −351 to nt +403) into a modified pREP10 episomal vector containing the luciferase reporter gene, the EBV replication origin (OriP) and encoding the nuclear antigen EBNA-1 (see Methods section for details). Indeed, several studies have reported that pREP-based episomal constructs display hallmarks of proper chromatin structure when transiently transfected into cells^40^,^41^,^42^,^43^. However, as the HTLV-1 5′-LTR contains the promoter for the majority of viral genes but complementary strand of the proviral genome encodes the HBZ protein which is regulated by an independent antisense transcriptional promoter located in the 3′-LTR, we cloned the complete HTLV-1 LTR in both the sense and antisense orientations with respect to the luciferase transcriptional unit, thereby generating the episomal plasmids referred to as pREP-LTRwt_sense-luc and pREP-LTRwt_antisense-luc, respectively. As controls, we also cloned, in both orientations, the HTLV-1 LTR in a pREP10 vector in which we deleted the EBV replication origin (OriP) and the nuclear antigen EBNA-1, thereby generating the non episomal plasmids referred to as pREP∆-LTRwt_sense-luc and pREP∆-LTRwt_antisense-luc, respectively. These deletions of the OriP and EBNA-1 regions should incapacitate the vector to adopt the native proper chromosomal structure. Efficient transcription and replication of the HTLV-1 genome require both the viral LTR and the virus-encoded transcriptional activator Tax. We therefore first compared Tax-mediated transactivation of the HTLV-1 promoter cloned in the episomal pREP10 reporter vector in the sense or antisense orientations upstream of the luciferase gene (pREP-LTRwt_sense-luc and pREP-LTRwt_antisense-luc) with the Tax transactivation of the non episomal pREP∆-LTRwt_sense-luc and pREP∆-LTRwt_antisense-luc reporter vectors (Fig. 5). To this end, we transiently cotransfected Jurkat T cells with these plasmids and increasing amounts of an expression vector for the viral transactivator Tax (pRSV-Tax) and the pRL-TK vector as an internal control. At 48 h post-transfection, luciferase activities were measured in cell lysates.

Results presented in Fig. 5a showed that Tax strongly transactivated all the sense reporter constructs in a dose-dependent manner despite variations in the transactivation amplitudes. Indeed, Tax transactivations of the episomal pREP-LTRwt_sense-luc construct ranged from 4.39- to 23.99-fold (Fig. 5, lanes 2 to 6), whereas Tax transactivations of the non episomal pREP∆-LTRwt_sense-luc luciferase activity were lower and ranged from 0.98- to 6.44-fold (Fig. 5, lanes 8 to 12). The antisense HTLV-1 LTR promoter was weakly transactivated by Tax when the LTR was cloned both in the episomal vectors (Fig. 5b, lane 2 to 6, ranged from 0.93- to 4.68-fold) and in the non episomal vectors (Fig. 5b, lane 8 to 12, ranged from 1.30- to 2.02-fold). Of note, these differences are not due to differences in Tax expression since the Tax mRNA levels were similar between the various dose responses (Supplemental Figure S1). These data clearly demonstrate the importance to study HTLV-1 transcriptional regulation in a chromatin context close to the *in vivo* situation and constitute the first direct demonstration of this critical issue of the nucleosomal environment for Tax-mediated transactivation of the HTLV-1 LTR.

In order to determine the relative contribution of each Sp1 binding site to the basal and Tax-mediated HTLV-1 LTR activity, the point mutations identified in EMSAs (Fig. 3) were introduced individually or in combinations in the context of the pREP-luc episomal luciferase reporter vector in both the sense and antisense orientations with respect to the luciferase transcriptional unit, thereby generating a serie of plasmids described in Supplementary Table S2.

**Figure 6:**
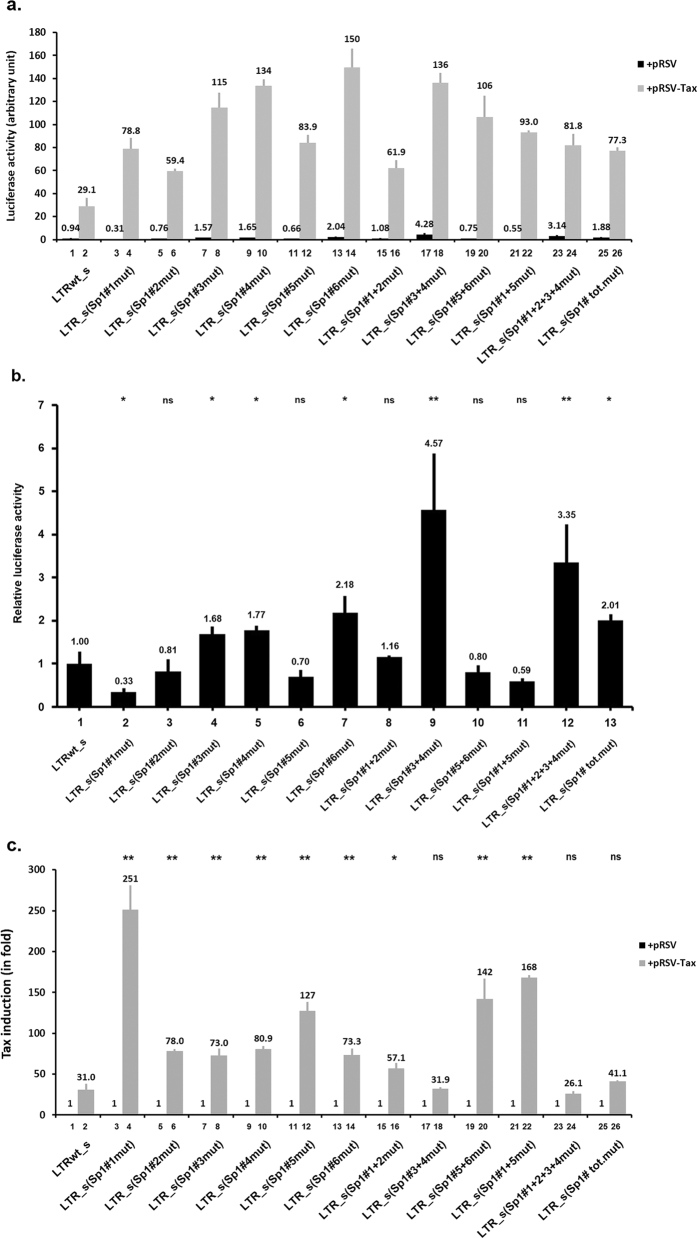
Effects of HTLV-1 Sp1-binding sites mutations on basal and Tax-transactivated 5′-LTR promoter activity. Using site-directed mutagenesis, we generated HTLV-1 sense LTR promoter luciferase episomal constructs containing 2-bp mutations abolishing Sp1 binding to the LTR Sp1 binding sites, alone or in combination (see Supplementary Table S2 for constructs description). These generated plasmids (600 ng of DNA) were cotransfected, with 400 ng of the pRSV empty vector (+pRSV) or with 400 ng of the Tax expression vector (+pRSV-Tax) into Jurkat T cells. Each transfection included 10 ng of the internal control *Renilla* luciferase plasmid, pRL-TK. Luciferase activities (Firefly and *Renilla*) and protein concentrations were measured in cell lysates 48 h after transfection. The results are expressed as luciferase_firefly_/luciferase_*Renilla*_/[protein] and presented as histograms indicating luciferase activity (arbitrary unit presented in (**a**)) of each reporter construct and relative to that measured with the pREP-LTRwt_sense-luc (LTRwt_s), which was arbitrarily assigned a value of 1, in the absence of the Tax transactivator (presented in (**b**)). Induction by Tax (in fold) with respect to the activity of the same reporter construct in the absence of Tax, which was arbitrarily assigned a value of 1 are presented in (**c**). Means and standard errors of the means from triplicate samples are represented. An experiment representative of three independent experiments is shown. ns corresponds to a p value > 0.05, *Corresponds to a p value < 0.05 and **to a p value < 0.01 in a Student’s t-test (in comparison with the construct LTRwt_s in absence (in (**b**)) or in presence (in (**c**)) of the viral Tax transactivator).

To assess the sense transcriptional regulatory function of the HTLV-1 LTR Sp1 motifs, we transiently cotransfected Jurkat T cells with the wild-type or the different mutated constructs in the absence or in the presence of the Tax expression vector (pRSV-Tax) and the pRL-TK as an internal control. At 48 h post-transfection, cells were lysed and assayed for luciferase activity (Fig. 6a).

In absence of Tax, results presented in Fig. 6b showed that both mutations in the R region Sp1 binding sites, Sp1#3 mut and Sp1#4 mut (Fig. 6b, lanes 4 and 5, respectively), resulted in a weak increase of the sense HTLV-1 LTR-driven basal luciferase gene expression (1.68-fold and 1.77-fold, respectively). The combinatory mutation Sp1#3 + 4 mut resulted in a strong increase (4.57-fold, Fig. 6b, lane 9), suggesting a cooperative repressive regulatory role of the R region Sp1 binding sites in the HTLV-1 sense promoter basal activity. Our results also showed an activator role of the Sp1#1 binding site in the sense HTLV-1 LTR promoter activity (Fig. 6b, lane 2) in agreement with results previously reported by Nyborg and coworkers^36^. Moreover, we showed that the Sp1#2 and Sp1#5 binding sites did not affect the basal sense HTLV-1 LTR promoter activity (Fig. 6b, lanes 3 and 6, respectively). These later data were in opposition with Yao’s^33^ and Okumura’s studies^35^, respectively. Interestingly, we observed an increase in luciferase activity of the pREP-LTR_sense (Sp1#6 mut)-luc compared to activity of the wild-type pREP-LTRwt_sense-luc construct (Fig. 6b, compare lanes 7 with lane 1), suggesting a negative *cis*-regulatory role of the Sp1#6 site in the sense basal HTLV-1 LTR promoter activity. Importantly, the pREP-LTR_sense (Sp1#1 + 2 + 3 + 4 mut)-luc and the pREP-LTR_sense (Sp1#tot.mut)-luc constructs exhibited a basal luciferase activity 3.35- and 2.01-fold higher than the activity of the wild-type vector containing the sense LTR (Fig. 6b, compare lanes 12 and 13, respectively, with lane 1), suggesting that all together the Sp1 binding sites act as negative *cis*-regulatory elements for the sense LTR-driven expression in the absence of Tax.

In presence of Tax, we observed a 31.0-fold Tax transactivation of the wild-type HTLV-1 LTR (Fig. 6c, lane 2). When we mutated all the Sp1 binding sites individually (Sp#1 mut, Sp#2 mut, Sp#3 mut, Sp#4 mut, Sp#5 mut or Sp#6 mut) or in combination (Sp1#1 + 2 mut, Sp1#5 + 6 mut, Sp1#1 + 5 mut), the sense LTR transactivation mediated by Tax increased (Fig. 6c, lanes 4, 6, 8, 10, 12, 14, 16, 20 and 22, respectively). Of note, this differences are not correlated to variation in the Tax mRNA levels (Supplemental Figure S2.a). These results suggest a repressive *cis-*regulatory role of the HTLV-1 Sp1 binding sites in Tax-regulated viral transcription.

Finally, we observed that the combined mutation of the Sp1#3 + 4 binding sites did not alter Tax transactivation (31.9-fold Tax transactivation for the pREP-LTR_sense (Sp1#3 + 4 mut)-luc construct compared to the 31-fold Tax transactivation of the wild-type pREP-LTRwt_sense-luc vector, Fig. 6c, lane 18 compared to lane 2).

Altogether, these results suggest that, the Sp1 binding sites located in the R region of the HTLV-1 promoter act as negative *cis*-regulatory elements for HTLV-1 LTR-driven basal sense gene expression. Moreover, the Sp1#3 and Sp1#4 binding sites do not seem to impede Tax-mediated transactivation of the LTR sense transcriptional activity since the Tax fold transactivations observed with the constructs of the wild-type and the mutant Sp#3 + 4 binding sites were similar.

**Figure 7:**
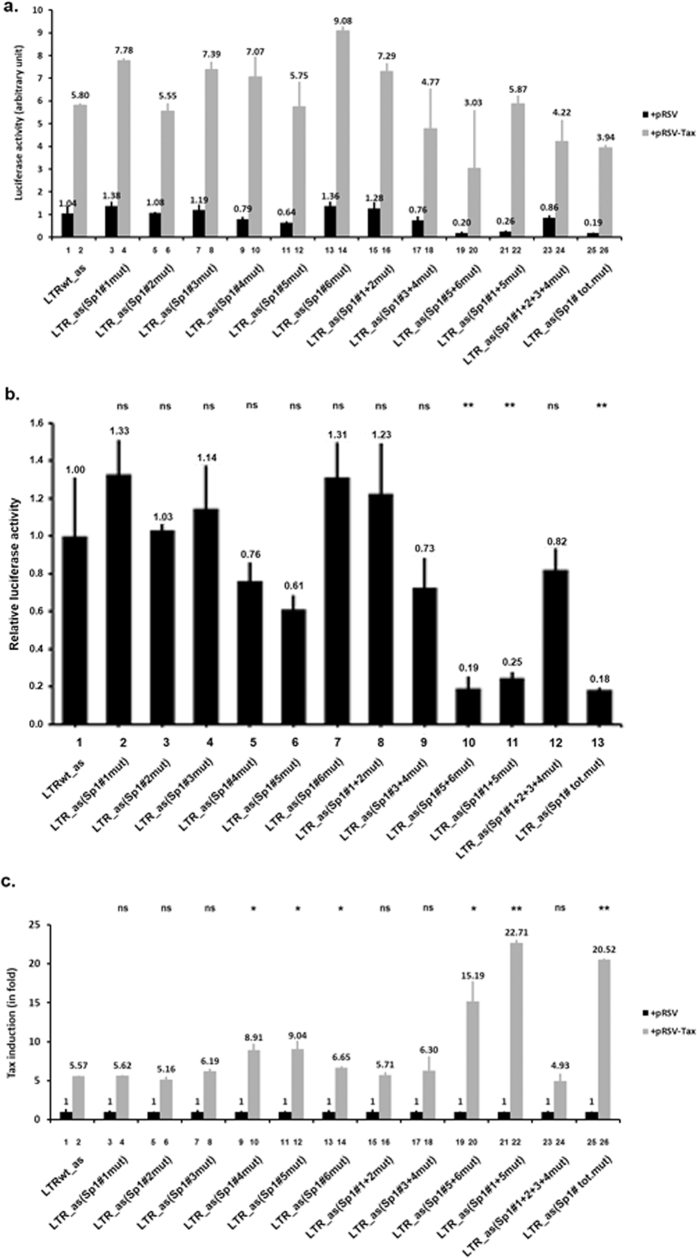
Effects of HTLV-1 Sp1-binding sites mutations on basal and Tax-transactivated 3′-LTR promoter activity. Using site-directed mutagenesis, we generated HTLV-1 antisense LTR promoter luciferase episomal constructs containing 2-bp mutations abolishing Sp1 binding to the LTR Sp1 binding sites, alone or in combination (see Supplementary Table S2 for constructs description). These generated plamsids (600 ng of DNA) were cotransfected, with 400 ng of the pRSV empty vector (+pRSV) or with 400 ng of the Tax expression vector (+pRSV-Tax) into Jurkat T cells. Each transfection included 10 ng of the internal control *Renilla* luciferase plasmid, pRL-TK. Luciferase activities (Firefly and *Renilla*) and protein concentrations were measured in cell lysates 48 h after transfection. The results are expressed as luciferase_firefly_/luciferase_*Renilla*_/[protein] and presented as histograms indicating luciferase activity (arbitrary unit presented in (**a**)). of each reporter construct and relative to that measured with the pREP-LTRwt_antisense-luc (LTRwt_as), which was arbitrarily assigned a value of 1, in the absence of the Tax transactivator (presented in (**b**)). Induction by Tax (in fold) with respect to the activity of the same reporter construct in the absence of Tax, which was arbitrarily assigned a value of 1 are presented in **c**. Means and standard errors of the means from triplicate samples are represented. An experiment representative of three independent experiments is shown. ns corresponds to a p value > 0.05, *Corresponds to a p value < 0.05 and **to a p value < 0.01 in a Student’s t-test (in comparison with the construct LTRwt_as in absence (in (**b**)) or in presence (in (**c**)) of the Tax transactivator).

We next assessed the transcriptional regulatory function of the HTLV-1 LTR Sp1 binding sites on HTLV-1 antisense transcription. We transiently cotransfected Jurkat T cells with the wild-type or different mutated constructs, in which the HTLV-1 LTR was cloned in the antisense orientation, and with the pRL-TK vector in the absence or presence of the Tax expression vector. At 48 h post-transfection, cells were lysed and assayed for luciferase activity (Fig. 7a).

In absence of Tax, results presented in Fig. 7b showed that the Sp1 binding sites individual mutations had no effect on antisense LTR basal activity (Fig. 7b, lanes 2, 3, 4, 5, 6 and 7). Importantly, the pREP-LTR_antisense (Sp1#5 + 6 mut)-luc construct exhibited a basal luciferase activity 5-fold ( = 1/0.19) lower than the activity of the wild-type vector pREP-LTRwt_antisense-luc containing the antisense LTR (Fig. 7b, compare lane 10 with lane 1), suggesting that together the Sp1#5 and Sp1#6 binding sites act as positive *cis*-regulatory elements for antisense LTR expression in the absence of Tax. We observed similar results for the pREP-LTR_antisense (Sp1#1 + 5 mut)-luc (4-fold repression of the measured luciferase activity, Fig. 7b, compare lane 11 with lane 1) and for the pREP-LTR_antisense (Sp1#tot.mut)-luc (6-fold repression of the measured luciferase activity, Fig. 7b, compare lane 13 with lane 1).

These results suggest that the Sp1 binding sites located at the U5 region of the HTLV-1 promoter (Sp1#5 and Sp1#6) are important for the antisense promoter activity, in agreement with Yoshida’s previous study^34^. Moreover, the combinatory mutation Sp1#3 + 4 mut did not alter the LTR antisense basal activity (Fig. 7b, compare lane 9 with lane 1).

In the presence of Tax, we observed a 5.57-fold Tax transactivation of the wild-type HTLV-1 LTR-driven antisense transcription (Fig. 7c, lane 2). This transactivation was not affected by individual mutations in the LTR Sp1#1, Sp1#2 and Sp1#3 binding sites (Fig. 7c, lanes 4, 6 and 8, respectively) or by the combined mutations Sp1#1 + 2 mut and Sp1#1 + 2 + 3 + 4 mut (Fig. 7c, lanes 16 and 24, respectively). In contrast, the individual mutations Sp1#4 mut, Sp1#5 mut, and Sp1#6 mut (Fig. 7c, lane 10, 12 and 14, respectively) and the combined mutations, Sp1#5 + 6 mut and Sp1#1 + 5 mut (Fig. 7c, lane 20 and 22, respectively) caused an increased Tax transactivation of the LTR antisense activity while the Tax mRNA levels were similar (Supplemental Figure S2.b). We obtained similar results for the pREP-LTR_antisense (Sp1#tot.mut)-luc construct (Fig. 7c, lane 26). These results suggest a negative *cis-*regulatory role of the HTLV-1 Sp1 binding sites in the Tax-regulated antisense transcriptional activity of the viral LTR. Interestingly, the combined mutation Sp1#3 + 4 mut did not alter Tax responsiveness of the HTLV-1 LTR (Fig. 7c, compare lane 18 with lane 2).

Taken together, these observations suggest that the two new Sp1 binding sites identified here are critical for the HTLV-1 LTR sense transcriptional activity, but not for the transcriptional activity of the LTR in the antisense orientation. Moreover, we showed that the integrity of all six HTLV-1 Sp1 binding sites is not necessary for Tax transactivation. Importantly, our study constitutes the first report evaluating point mutations in HTLV-1 promoter transcription factor binding sites in the context of proper chromatin organization.

### Mutations in the Sp1#3 and Sp1#4 binding sites of the HTLV-1 promoter impair Sp1 *in vivo* recruitment to the R region of the LTR

**Figure 8:**
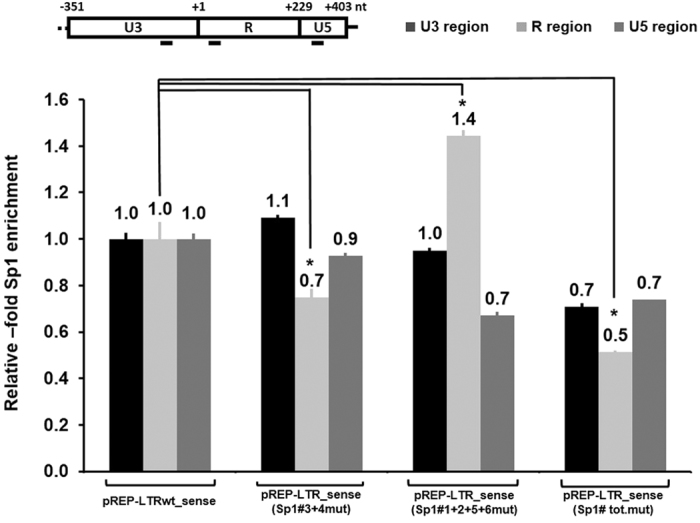
Mutations in the Sp1-binding sites located in the R region of the HTLV-1 promoter affect the *in vivo* recruitment of Sp1 to the 5′-LTR region. HEK 293 T cells were transiently transfected with the wild type pREP-LTRwt_sense-luc, the pREP-LTR_sense (Sp1#3 + 4 mut)-luc, the pREP-LTR_sense (Sp1#1 + 2 + 5 + 6 mut)-luc or the pREP-LTR_sense (Sp1#tot.mut)-luc. At 24 h post-transfection, cells were cross-linked for 10 min at room temperature with 1% formaldehyde. Pellets were sonicated to obtain DNA fragments of an average of 200 bp. Chromatin immunoprecipitations were performed with specific antibody directed against Sp1. To test aspecific binding to the beads, a purified IgG was used as a control for immunoprecipitation. Quantitative PCR reactions were performed with oligonucleotide primers hybridizing in the U3 region (from nt −216 to −299 where Sp1#1 and Sp1#2 are located), in the R region (from nt + 6 to + 111 where Sp1#3 and Sp1#4 are located) and in the U5 region (from nt + 247 to + 353 where Sp1#5 and Sp#6 are located) of the HTLV-1 promoter. Specific Sp1 enrichment was calculated relative to the value obtained with the wild type pREP-LTRwt_sense-luc. Means and standard errors of the means from experiment representative of two independent ChIP assays are shown. *Corresponds to a p value < 0.05 in a Student’s t-test.

The recruitment of the transcription factor Sp1 to the HTLV-1 promoter in infected cell lines has been previously reported^33^,^34^,^36^. In order to determine whether endogenous Sp1 transcription factor is recruited *in vivo* to the Sp1#3 and Sp1#4 binding sites identified here, we performed chromatin immunoprecipitationn (ChIP) assays in transfected HEK 293 T cell line. The cells were transiently transfected with the wild-type LTR reporter plasmid (pREP-LTRwt_sense-luc), the Sp1#3 + 4 mutated construct, the Sp1#1 + 2 + 5 + 6 mutated construct or the total Sp1 binding sites mutated construct. Twenty-four hours post-transfection, crosslinked chromatin from these cells was sheared to approximately 200 bp and immunoprecipitated with a specific antibody directed against Sp1 or with purified IgG as a control. Three different primer pairs were designed in the HTLV-1 promoter to study: 1) the U3 region where Sp1#1 and Sp1#2 are located, 2) the R region where Sp1#3 and Sp1#4 are located, and 3) the U5 region where Sp1#5 and Sp1#6 are located, respectively. As shown in Fig. 8, Sp1 recruitment at the R region of the HTLV-1 promoter was decreased by mutations introduced in the Sp1#3 and Sp1#4 binding sites but increased by mutations introduced in the other Sp1 binding sites (pREP-LTR_sense-Sp1#1 + 2 + 5 + 6 mut-luc), while similar levels of Sp1 protein were present in the whole cellular extracts (Supplemental Figure S3). Interestingly, Sp1 recruitment at the U3 and U5 regions was similar when we used the wild-type or the Sp1#3 + 4 mutated LTR constructs. Finally, we showed a global decrease in Sp1 recruitment at the HTLV-1 promoter when all the Sp1 binding sites region were mutated.

These results indicate that Sp1 is recruited *in vivo* to the Sp1#3 and Sp1#4 binding sites of the HTLV-1 promoter in the context of transiently transfected episomal reporter constructs, thereby confirming the physiological relevance of these Sp1 binding sites.

## Discussion

After integration into the host cellular genome, the HTLV-1 provirus is transcriptionally regulated by cellular transcription factors such as the ubiquitously expressed Sp1 factor. Interestingly, transcriptional regulation by Sp1 has already been reported in mammalian retroviruses such as the Moloney Murine Leukemia Virus (Mo-MLV) and its closely related derivative Moloney Murine Sarcoma Virus (Mo-MSV). The presence of a single Sp1 binding site in a variant of the Mo-MSV is sufficient to activate the basal transcription level of the LTR in embryonal carcinoma stem cells^44^. Moreover, the expression of the Human Endogenous Retrovirus (HERV) is upregulated by the Sp1 and Sp3 transcription factors^45^,^46^. Finally, Sp1 is also important for gene regulation of HIV-1 where it plays repressive and activating roles^46^,^47^,^48^,^49^. The three HIV-1 LTR Sp1 binding sites located in the U3 region play essential roles in regulation of basal and Tat-transactivated HIV-1 LTR activities^50^. It has also been demonstrated that the HIV-1 Sp1 binding site located in the leader sequence regulates (untranslated transcribed region downstream of the transcription start site of the 5′-LTR of HIV-1) basal transcription from the 5′-LTR promoter^51^,^52^. Moreover, the *pol* gene of HIV-1 contains a Sp1 binding site implicated in the transcriptional enhancer activity of the intragenic *cis-*regulatory region^47^,^48^.

Therefore, in this report, we analyzed *in silico* the nucleotide sequence of the HTLV-1 promoter and identified two new Sp1 binding sites located in the R region of the viral LTR (referred to as the Sp1#3 and Sp1#4 binding sites). We demonstrated by competition and supershift EMSAs that the transcription factor Sp1 bound in a sequence-specific manner to the Sp1#3- and Sp1#4-containing sequences but Sp3 bound preferentially the Sp1#4-containing sequence. We also demonstrated by ChIP experiments the *in vivo* recruitment of transcription factor Sp1 to the Sp1#3 and Sp1#4 binding sites and a decrease in Sp1 recruitment when these two Sp1 binding sites were mutated, thereby confirming the physiological relevance of these two new Sp1 binding sites. Further functional characterization of these Sp1 binding sites revealed that ectopically expressed Sp1 had a positive effect on a luciferase reporter plasmid containing three tandem copies of the HTLV-1 Sp1#3 + 4 motif cloned upstream of the heterologous promoter TK in *Drosophila* SL2 cell line. Interestingly, we observed that the Sp1 stimulatory effect was dependent on the Sp1#3 + 4 motif orientation cloned upstream on the TK promoter. Indeed, the antisense motif was more transactivated by Sp1 than the sense motif. These activator effects were in contrast with our results presented in Figs 6 and 7 (Fig. 6a, lane 9 and Fig. 7a, lane 9), but it is important to note that the biological context was different because the non-episomal minimal TK-promoter luciferase reporter construct allows to study the potential role of only the two newly identified HTLV-1 Sp1#3 + 4 binding sites in a different context that the complete LTR in a *Drosophila* cell line devoid of Sp factors.

Several other Sp1 binding sites have been previously identified in either the 5′-LTR or 3′-LTR of the HTLV-1 genome^33^,^34^,^35^,^36^ (see Fig. 1b). In the present report, we established, for the first time, a ranking of all the Sp1 binding sites of the HTLV-1 promoter with respect to their affinity for Sp1 in the same experimental settings. We showed that the Sp1#1 and Sp1#5 binding sites were the strongest in terms of affinity for Sp1 protein, the Sp1#6 and Sp1#4 binding sites had similar binding affinity and the Sp1#2 and Sp1#3 binding sites exhibited the lowest affinity. So far, the function of the HTLV-1 Sp1 binding sites has never been studied in a physiologically relevant context. Indeed, the previous findings were based on transient transfection assays using either fragments of the viral LTR^33^,^34^,^36^, cell lines which are not natural targets for HTLV-1^33^,^36^ or non episomal plasmids^33^,^34^,^35^,^36^ which do not reconstitute a native nucleosomal organization. In order to address the function of HTLV-1 Sp1 binding sites in more physiological conditions, we here cloned the HTLV-1 LTR in a pREP-based episomal vector displaying proper chromatin-like structure^40^,^41^,^42^,^43^. We compared the Tax-mediated transactivation of the HTLV-1 promoter cloned in an episomal vector (pREP) or a non-episomal vector (pREPΔ) using transient transfection assays in Jurkat T cells and showed the importance of the nucleosomal environment for the Tax transactivation of HTLV-1 gene expression. Notably, since the 3′-LTR contains the *hbz* promoter, we also cloned the HTLV-1 LTR in the antisense orientation upstream of the luciferase gene. Our results showed that the antisense LTR was also transactivated by Tax, which is in agreement with the findings of Yoshida *et al*. who have previously reported that the minimal promoter of *hbz* gene is inducible by Tax^34^.

Next, we compared the effect of Sp1 binding sites on the HTLV-1 promoter activities using the same experimental settings as described above. Interestingly, we showed that the individual mutations of the Sp1#3 or Sp1#4 sites, as well as the combined mutations abolishing Sp1 binding to both the Sp1#3 and Sp1#4 binding sites together, exhibited repressor effects on the LTR sense transcriptional activity but had no effect on the LTR antisense transcriptional activity, suggesting that these Sp1#3 and Sp1#4 sites act as negative *cis*-regulatory elements of HTLV-1 gene expression. Moreover, the combined mutation Sp1#3 + 4 mut caused the highest increase in HTLV-1 sense LTR-driven gene expression compared to all the other individual and combined mutations we tested. These results, addressing the function of the HTLV-1 LTR Sp1 binding sites in a nucleosomal context, are in agreement with the work of Yoshida *et al*.^34^ who have described that the Sp1 binding sites located in the HTLV-1 *hbz* promoter (referred to as Sp1#5 and Sp1#6 binding sites) are critical for HBZ promoter activity. Indeed, we showed here that these sites were, among all the HTLV-1 Sp1 sites tested, the only *cis*-acting elements positively regulating the antisense LTR activity. However, contradictions with some previous studies describing the role of some HTLV-1 Sp1 binding sites^33^,^34^,^35^,^36^ exist. First, we demonstrated here that the Sp1#2 mutation did not affect LTR basal expression. In contrast, Yao *et al*.^33^ have previously shown that transcription factors Sp1 and Sp3 affect basal and Tax-transactivated activity of an HTLV-1 LTR containing mutations in the Sp#2 binding site. Second, our results addressing the role of the Sp1#5 binding site which is located in the U5RE region are in contradiction with the Okumura’s study^35^. These later authors have shown that U5RE (containing only the Sp1#5 site and not the Sp1#6 site) exerts a repressive effect on basal LTR-mediated expression and that a single point mutation in this region diminishes this U5RE repressive effect, whereas in our experimental settings the Sp1#5 site had no effect on basal HTLV-1 promoter activity cloned in sense orientation. Overall, these discrepancies between our present work and previous studies could be explained by differences between our experimental settings and those used in the previous studies, such as the cell lines used, episomal *versus* non episomal reporter vectors, full-length *versus* truncated LTRs cloned in the reporter vectors. Importantly, in contrast to these previous studies, we here performed, for the first time, a functional comparison of all the HTLV-1 Sp1 binding sites in the more physiological nucleosomal context of an episomal reporter vector and we evaluated the contribution of each Sp1 site separately to both sense and antisense transcriptional activities of the HTLV-1 LTR.

Interestingly, we observed that the sense LTR transactivation mediated by Tax increased when the Sp1 binding sites were mutated individually or in combination, suggesting a repressive *cis-*regulatory role of the HTLV-1 Sp1 binding sites in Tax-regulated viral transcription. These data suggest that the Sp1 binding site mutations could allow an increased Tax-CREB/ATF recruitment to the HTLV-1 promoter by decreasing the steric hindrance due to recruitment of both CREB/ATF and Sp1 (or by decreasing the recruitment of a repressor). These results are in agreement with the Yao’s study^33^. Indeed, these authors have reported that Sp1 competes with CREB for binding to the CRE-3 motif due to the physical proximity of the Sp1#2 binding site and the CRE-3 motif, while, in the presence of Tax, Sp1 forms a multiprotein complex with CREB/ATF and Tax^33^.

The HTLV-1 Sp1#3 and Sp1#4 binding sites identified here in the LTR R region acted as negative *cis*-regulatory elements. The Sp1 and Sp3 factors share the same DNA recognition motif and Sp3 is more often associated with repression of Sp1-mediated transcriptional activation depending on the sequence context, the number of Sp1 binding sites and the availability of specific coactivators, corepressors and/or other transcription factors^20^,^21^,^53^. Therefore, we cannot exclude that the two new Sp1 binding sites would be bound *in vivo* by Sp3 and that this would lead to repression of basal HTLV-1 transcription. Sp3-mediated transcriptional repression can result from competition with Sp1 for the same DNA binding site or from steric hindrance between repressors and positively acting transcriptional factors^20^,^21^,^50^. In our future experiments, we plan to study the involvement of different Sp transcription factors, with a special emphasis on Sp3, in HTLV-1 transcriptional regulation and the potential cross-talk between these Sp factors.

In conclusion, we identified two new functional Sp1 binding sites located in the R region of the HTLV-1 LTR. We provide evidence that these two sites exhibit repressive activity exclusively on the regulation of viral sense transcription and we are currently studying their effect on the HTLV-1 replication cycle. Moreover, the exhaustive characterization of all six LTR Sp1 binding sites in the chromatin-associated regulation of both viral sense and antisense LTR transcriptions bring an additional factor in an already complex network of regulators affecting the level of HTLV-1 gene expression.

## Methods

### Cell lines and cell culture

The T-lymphoid cell line Jurkat^54^ and the human embryonic kidney cell line HEK 293 T^55^ were obtained from the AIDS Research and Reference Reagent Programme (National Institute of Allergy and Infectious Diseases [NIAID], National Institutes of Health [NIH]). The Jurkat cells were maintained in RPMI 1640-Glutamax medium (Life Technologies) supplemented with 10% fetal bovine serum (FBS) and the HEK 293 T cells in Dulbecco’s modified Eagle’s-Glutamax medium (Life Technologies) supplemented with 10% FBS and 1 mM sodium pyruvate. The *Drosophila* SL-2 cell line^56^ was cultured in Schneider’s *Drosophila* medium (Life Technologies) supplemented with L-glutamine and with 10% FBS. All media also contained 50 U of penicillin/ml and 50 μg of streptomycin/ml (Life Technologies). Jurkat and HEK 293 T cells were grown at 37 °C in a 95% air-5% CO_2_ humidified atmosphere and SL-2 cells were grown at 28 °C under normal atmosphere.

### Bioinformatic Analyses

Using database associated search tools (Alibaba 2.1, TF Search, TESS and Motif), the HTLV-1 LTR sequence was scanned for the presence of putative *cis*-acting regulatory elements identical with or similar to the motifs registered in TRANSFAC (http://www.gene-regulation.com).

### Plasmids constructs

PCR was used to amplify the HTLV-1 LTR region from the molecular clone K30 described by Zhao *et al*.^57^ (received from the AIDS Research and Reference Reagent Program, NIAID, NIH). The first nucleotide (nt) of the R region was considered as nt + 1. The 5′ primer oligonucleotide corresponding to nt −354 to −331 contained an added *BglII* site (in bold) at the 5′ end (5′-GA**AGATCT**TGAACAATGACCATGAGCCCCAA-3′). The 3′ primer oligonucleotide corresponding to nt + 355 to + 374 contained an added *BglII* site (in bold) at the 5′ end (5′-GA**AGATCT**TCTTGTGTACTAAGTTTCTCTCCTGGAG-3′). Amplification reaction was conducted with 100 ng of template plasmid DNA as specified in the protocol provided with the high fidelity *Pfu* DNA polymerase (Stratagene) with a DNA thermal cycler 480 (PerkinElmer Life Sciences). The *BglII*-restricted PCR fragment was cloned in the two orientations in the pGL3-BASIC and in the previously described pREP-luc^58^ digested with *BglII.* The resulting plasmids were designated pGL3-LTRwt_sense-luc and pGL3-LTRwt_antisense-luc, and pREP-LTRwt_sense-luc and pREP-LTRwt_antisense-luc, respectively.

To estimate the importance of proper chromatin formation, we have constructed the pREP-LTRwt-luc plasmids deleted from the Epstein-Barr virus replication origin and from nuclear antigen (encoded by the EBNA-1 gene). To obtain the pREP∆-luc, pREP∆-LTRwt_sense-luc and pREP∆-LTRwt_antisense-luc, the plasmids pREP-luc, pREP-LTRwt_sense-luc and pREP-LTRwt_antisense-luc, respectively, were digested by *XhoI* and *AvrII*, 3′ blunt-ended with DNA Polymerase I Large (Klenow) Fragment and self-ligated with T4 DNA ligase.

The pGL3-LTRwt_sense-luc construct was used as a substrate for mutagenesis (by the QuickChange Site-directed Mutagenesis method, Stratagene). Different mutations were generated with pairs of mutagenic oligonucleotide primers (described in the Supplementary Table S1). Mutated constructs were fully sequenced after identification and were described in the Supplementary Table S2. Constructs containing combinations of mutations were also generated by site-directed mutagenesis by series of successive steps of single mutagenesis.

All theses mutated constructs were digested by *BglII* and the LTR fragments were cloned in both orientations in the pREP-luc digested with *BglII*. The resulting plasmids were described in the Supplementary Table S2.

The expression vector coding for the HTLV-1 transactivator Tax (pRSV-Tax) and the empty pRSV vector were kindly provided by Dr. Françoise Bex (ULB, Brussels, Belgium). The Sp1 expression vector (pPacSp1) was kindly provided by Dr Guntram Suske.

The pTK-luc reporter construct contains the luciferase gene under the control of the Herpes Simplex Virus thymidine kinase (TK) minimal promoter and was generated by subcloning the *XmaCI-XhoI* fragment from the pGL2-TK^59^ into the *XmaCI-XhoI*-restricted pGL3-BASIC vector.

The p(Sp1#3 wt + 4 wt)_3_senseTK-luc and p(Sp1#3 wt + 4 wt)_3_antisenseTK-luc were generated by inserting upstream of the TK promoter an oligonucleotide corresponding to three copies of the Sp1#3 wt and Sp1#4 wt binding sites (Custom genes Method, Eurogentec, see Supplementary Table S3 for sequences) in forward and reverse orientations into *Sma*I-digested pTK-luc. We obtained similarly the two mutated versions, p (Sp1#3 mut + 4 mut)_3_senseTK-luc and p(Sp1#3 mut + 4 mut)_3_antisenseTK-luc.

### Transient transfections and luciferase assays

SL-2 cells were transiently transfected using FuGENE™HD (Promega) according to the manufacturer’s protocol. Briefly, one day before transfection, cells were seeded at a density of 3 × 10^4^ cells/well in 96-well plates in 200 μl of supplemented medium. FuGENE™HD was added directly to an aliquot of 10 μl of an Opti-MEM/DNA (200 ng) mixture in microcentrifuge tubes. The mixture was incubated for 15 min at room temperature and, finally added to each well. As an internal control for transfection efficiency, all transfection mixtures contained the pRL-TK vector, in which a cDNA encoding the *Renilla* luciferase is under the control of the HSV TK promoter region (pGL4.74, Promega). Twenty-four hours after transfection, cells were lysed and assayed for luciferase activities. Firefly luciferase activities derived from the HSV TK promoter were normalized with respect to the *Renilla* luciferase activities by using the Dual-Glo^®^ luciferase assay system (Promega).

Jurkat T cells were transiently transfected using JetPEI™ (Polyplus-transfection) according to the manufacturer’s protocol. Briefly, cells were seeded at a density of 5 × 10^5^ cells/well in 12-well plates in 1 ml of supplemented medium. For each well, 600 ng of DNA were diluted into 50 μl of 150 mM NaCl. The transfection reagent JetPEI™ (3.2 μl/well) was diluted into 150 mM NaCl (50 μl/well). An aliquot of 50 μl of this JetPEI™/NaCl solution was added to the 50 μl DNA solutions, and the JetPEI™/NaCl/DNA mixture incubated for 15 min at room temperature and added dropwise to each well. All transfection mixtures contained the pRL-TK vector as an internal control for transfection efficiency. Two hours after transfection, 500 μl of supplemented medium was added to each well. Forty-eight hours post-tranfection, cells were lysed and assayed for luciferase activities. Firefly luciferase activities derived from HTLV-1 promoter were normalized with respect to the *Renilla* luciferase activities by using the Dual-Luciferase^®^ Reporter Assay System (Promega), and to protein concentrations using the Bradford quantification method^60^.

### Chromatin immunoprecipitation assays *in vivo*

HEK 293 T cells were seeded at a density of 4 × 10^6^ cells/dish in 100-mm diameter dishes in 25 ml of supplemented medium and transfected using the FuGENE™6 (Promega) according to the manufacturer’s protocol with the indicated vectors. At 24 h post-transfection, cells were cross-linked for 10 min at room temperature with 1% formaldehyde and unreacted formaldehyde was quenched by addition of TRIS/glycine (125 mM final concentration). Cells were washed twice with phosphate buffered saline PBS, lysed in ChIP lysis buffer (Millipore) and sonicated (Bioruptor sonicator, Diagenode) to obtain DNA fragments of an average size of 200–400 bp. Chromatin immunoprecipitation assays were performed using the ChIP assay kit (EZ ChIP technology, Millipore) with an antibody directed against Sp1 (Millipore, 17–601). To test aspecific binding, a purified IgG was used as a control for immunoprecipitation (Vector Laboratories, I-1000). Quantitative Real Time PCR reactions were performed with the MESA GREEN qPCR MasterMix (Quanta). Relative quantification using standard curve method was performed for each primer pair, and 96-well Optical Reaction plates were read in a StepOnePlus PCR instrument (Applied Biosystems). Fold enrichments were calculated as percentages of input values following this formula: Immunoprecipitated DNA/total DNA (%). Primer sequences used for quantification in the U3 region (FW: 5′- CCCATTTCCTCCCCATGTT -3′; RV: 5′- TGCGTGCCATGAAAAGTTTT-3′), in the R region (FW: 5′- CTCGCATCTCTCCTTCACG- 3′; RV: 5′-ACGCAGTTCAGGAGGCAC-3′) and in the U5 region (FW: 5′- GTTCTGCGCCGCTACAG - 3′; RV: 5′- CTCCGAGCCAACGGAGT-3′) were designed using the software Primer express 2.0 (Applied Biosystems).

### Electrophoretic mobility shift assays

Nuclear extracts from Jurkat T cells were prepared using a protocol described by Dignam *et al*.^61^. The DNA sequences of the coding strand of the wild-type and mutated versions of the Sp1#1, Sp1#2, Sp1#3, Sp1#4, Sp1#5, Sp1#6 and consensus Sp1 binding sites used in this study are listed in the Supplementary Table S1. The various lengths of these oligonucleotides, which result from design constraints, may account for the different numbers of complexes observed with the different probes. EMSAs were performed as described previously^39^. Briefly, nuclear extracts (10 μg of protein) were first incubated for 10 min in the absence of probe and specific competitor DNA in a 16 μl reaction mixture containing 2 μg of DNase-free BSA (GE Healthcare), 2 μg of poly (dI-dC) (GE Healthcare) as non-specific competitor DNA, 50 μM ZnCl_2_, 0.25 mM DTT, 20 mM HEPES (pH 7.3), 60 mM KCl, 1 mM MgCl_2_, 0.1 mM EDTA and 10% (v/v) glycerol. 30,000 cpm of double-stranded probe (80–100 fmol) were then added to the mixture with or without a molar excess of an unlabeled specific DNA competitor, and the mixture incubated 20 min at room temperature. Samples were subjected to electrophoresis on 6% polyacrylamide gels at 120 V for 3 h in 1x TGE buffer [25 mM Tris-acetate (pH 8.3), 190 mM glycine and 1 mM EDTA]. Gels were dried and autoradiographed for 24–48 h at −80 °C. For supershift assays, antibodies (from Santa Cruz) against Sp1 (SC-59 X), Sp2 (SC-643 X), Sp3 (SC-644 X), Sp4 (SC-13019 X) or against a purified rabbit immunoglobulin (IgG; SC-2027) were added to the reaction mixture and incubated for 30 min at room temperature before the addition of the radiolabelled probe.

### Statistical analysis

Data sets from transfection experiments containing multiple comparisons (Figs 4 and 5) were analyzed using a two-way ANOVA test followed by paired comparisons between samples (Tukey’s test). Data sets from transfection experiments containing comparisons to the same reference condition (Figs 6 and 7) and from ChIP experiments were analyzed using a two-tailed unpaired T test. The threshold of statistical significance α was set at 0.05. Analyses were performed using Prism version 6.0 (GraphPad software).

## Additional Information

**How to cite this article:** Fauquenoy, S. *et al*. Repression of Human T-lymphotropic virus type 1 Long Terminal Repeat sense transcription by Sp1 recruitment to novel Sp1 binding sites. *Sci. Rep.* **7**, 43221; doi: 10.1038/srep43221 (2017).

**Publisher's note:** Springer Nature remains neutral with regard to jurisdictional claims in published maps and institutional affiliations.

## Supplementary Material

Supplementary Information
