# Supplementary material for: Repression of Human T-lymphotropic virus type 1 Long Terminal Repeat sense transcription by Sp1 recruitment to novel Sp1 binding sites
Source: Sci Rep. 2017 Mar 3;7:43221. doi: 10.1038/srep43221 (PMC5335701; doi:10.1038/srep43221)
Supplement: Supplementary Information [file srep43221-s1.doc]

**Repression of Human T-lymphotropic virus type 1 Long Terminal Repeat sense transcription by Sp1 recruitment to novel Sp1 binding sites**

Sylvain FAUQUENOY^1#^, Gwenaëlle ROBETTE^1§#^, Anna KULA^1^, Caroline VANHULLE^1^, Sophie BOUCHAT^1^, Nadège DELACOURT^1^, Anthony RODARI^1^, Céline MARBAN^2^, Christian SCHWARTZ^3,4^, Arsène BURNY^5^, Olivier ROHR^3,4¶^, Benoit VAN DRIESSCHE^1¶^ and Carine VAN LINT^1¶^*

^1^Service of Molecular Virology, Department of Molecular Biology (DBM), Université Libre de Bruxelles (ULB), Rue des Professeurs Jeener et Brachet 12, 6041 Gosselies, Belgium

^2^Biomaterials and Bioengineering, Inserm UMR 1121, Faculty of Dentistry, University of Strasbourg, France.

^3^Institut Universitaire de Technologie Louis Pasteur, University of Strasbourg, Schiltigheim, France.

^4^Laboratory of Dynamic of Host-Pathogen Interactions (DHPI), EA7292, University of Strasbourg, Strasbourg, France.

^5^Laboratory of Experimental Hematology, Institut Jules Bordet, Université Libre de Bruxelles (ULB), Brussels, Belgium.

^§^Present address : BePharbel Manufacturing, Rue du Luxembourg, Coucelles, Belgium.

^#^Both authors equally contribute to this work.

^¶^ These three authors equally contribute to this work.

*Correspondence and requests for materials should be addressed to C.V.L (email: cvlint@ulb.ac.be)

**
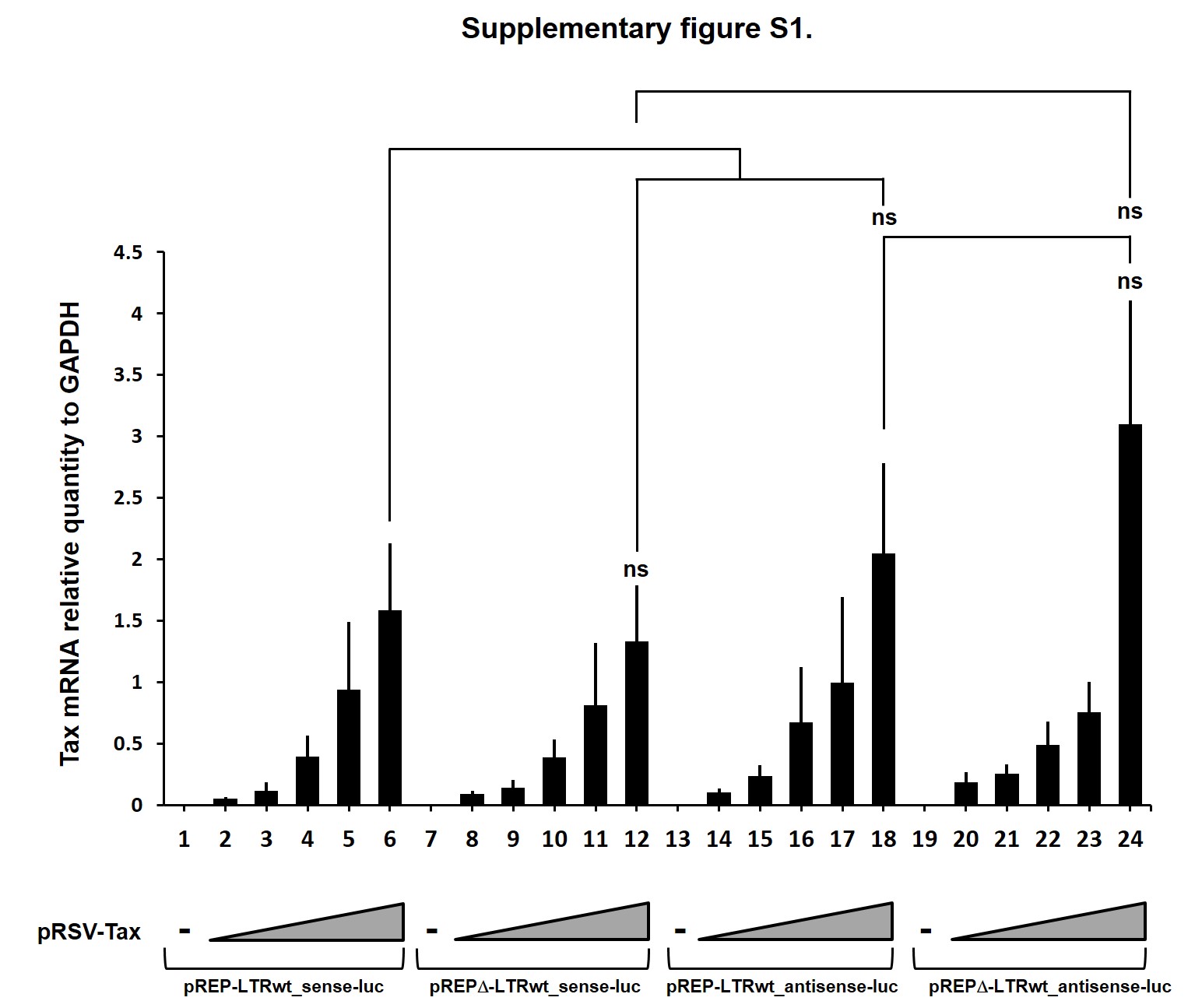
**

**Supplementary figure S1. Tax mRNA relative quantity normalized to GAPDH mRNA.** Total RNA from 1.10^6^ transfected cells was extracted using TRIzol (Life Technologies) followed by treatments with turbo DNase (Life Technologies) according to the manufacturer’s protocol. Retrotranscription reactions were performed with PrimeScript RT-PCR Kit (TaKaRa) using 200 ng of RNA and oligo(dT) oligonucleotides (for Tax and GAPDH genes). cDNA was then quantified by real-time PCR using the SYBR Premix Ex Taq (TaKaRa). Relative quantification using ∆∆Ct method was performed with 96-well Optical Reaction plates read in a StepOnePlus PCR instrument (Applied Biosystem). Primer sequences used for quantification of GAPDH (FW: 5’-GCCCCCGGTTTCTATAAATTG-3’ ; RV: 5’-AGAAGATGCGGCTGACTGTC-3’) and Tax (FW: 5’-ACCAATGTTCCCTACAAGCGA-3’ ; RV: 5’-TCGGGTAGAATTATTAGGGCC-3’) were designed using the software Primer express 2.0 (Applied Biosystems). The results are expressed as TAX mRNA level compared to GAPDH mRNA level. ns corresponds to a p value >0.05 in ANOVA test.


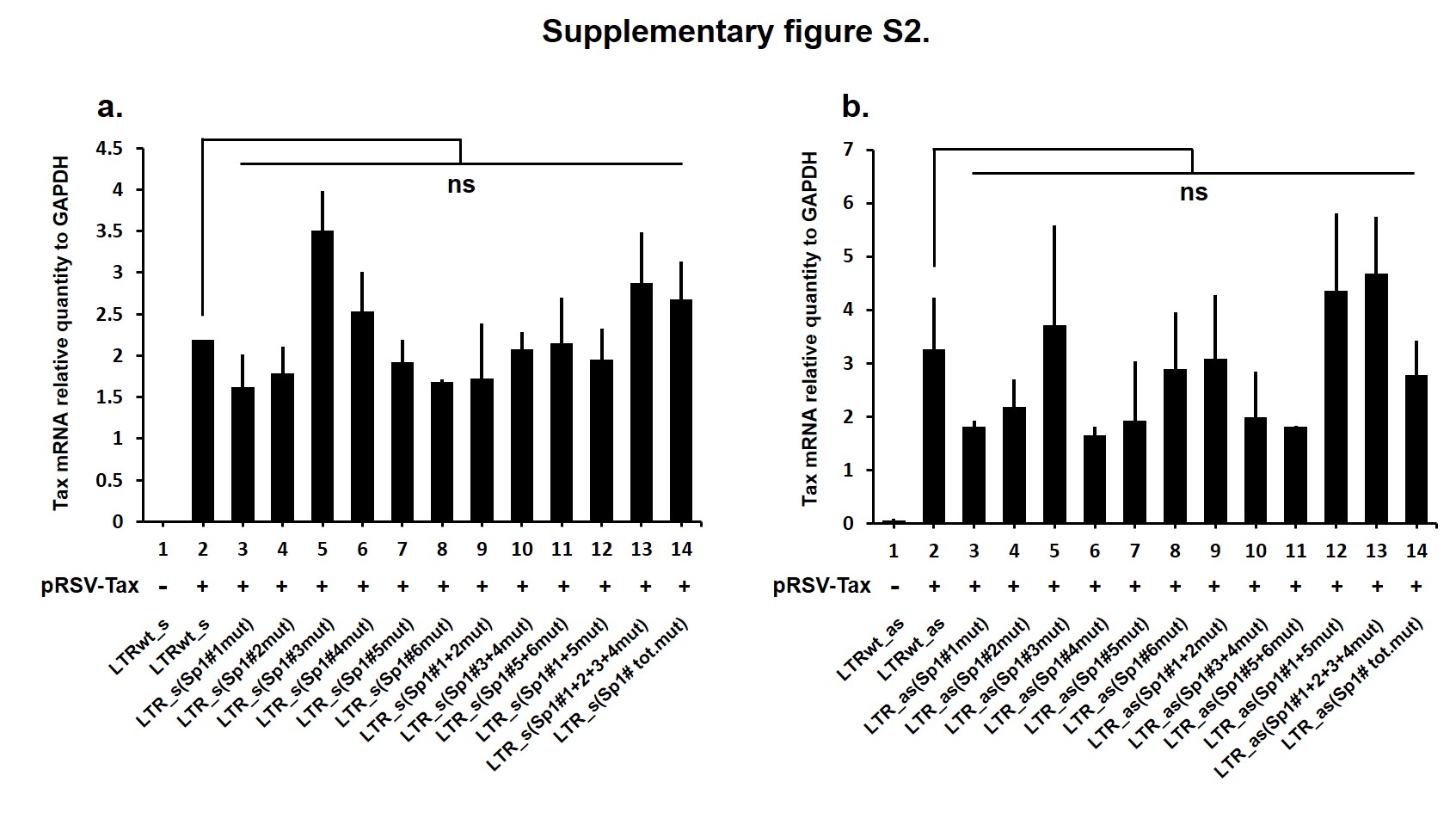


**Supplementary figure S2. Tax mRNA relative quantity normalized to GAPDH mRNA.** Total RNA from 1.10^6^ transfected cells was extracted using TRIzol (Life Technologies) followed by turbo DNAse (Life Technologies) according to the manufacturer’s protocol. Retrotranscription reactions were performed with PrimeScript RT-PCR Kit (TaKaRa) using 200 ng of RNA and oligo(dT) oligonucleotides (for Tax and GAPDH genes). cDNA was then quantified by real-time PCR using the SYBR Premix Ex Taq (TaKaRa). Relative quantification using ∆∆Ct method was performed with 96-well Optical Reaction plates read in a StepOnePlus PCR instrument (Applied Biosystem). Primer sequences used for quantification of GAPDH (FW: 5’-GCCCCCGGTTTCTATAAATTG-3’ ; RV: 5’-AGAAGATGCGGCTGACTGTC-3’) and Tax (FW: 5’-ACCAATGTTCCCTACAAGCGA-3’ ; RV: 5’-TCGGGTAGAATTATTAGGGCC-3’) were designed using the software Primer express 2.0 (Applied Biosystems). The results are expressed as TAX mRNA level compared to GAPDH mRNA level. ns corresponds to a p value >0.05 in ANOVA test.

**
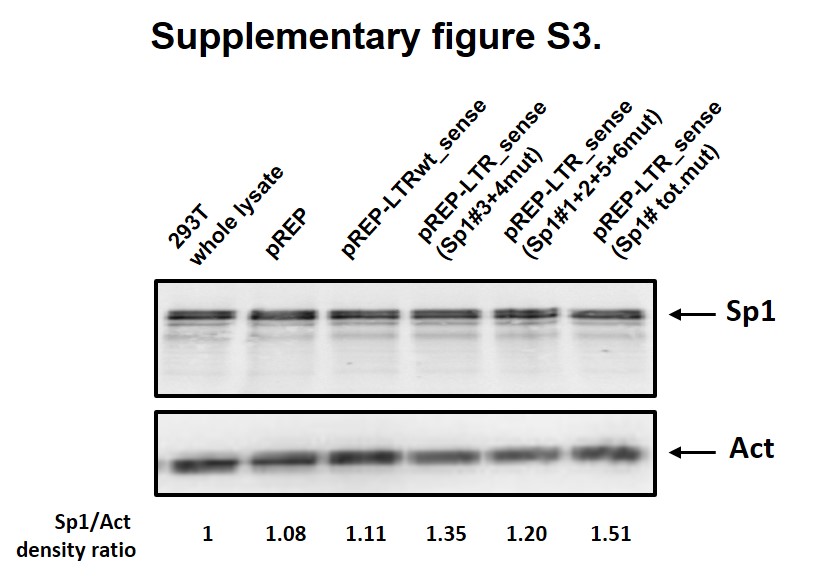
**

**Supplementary figure S3. Western blotting analysis of ChIP experiments.** 293T cells were transiently transfected with the indicated vectors and whole extracts were prepared using RIPA lysis buffer. Western blotting were performed with 10 μg of the total protein extracts. The immunodetection was assessed using antibodies targeting Sp1 (Millipore, 17-601) or actin (Sigma, A2066) as loading control. Ratios of the density of the bands corresponding to Sp1 and actin were calculated with the software ImageJ (National Institutes of Health, NIH) and a value of 1 was arbitrarily assigned to the value obtained with the 293T whole extracts.

**Supplementary Table S1. Oligonucleotides primers corresponding to LTR Sp1 binding sites** (Sp1 site is underlined on the coding strand primer and mutations are highlighted in bold)**.**

| **Numbers** (forward/reverse) | **Name** | **Sequence of the forward oligonucleotide** |
| --- | --- | --- |
| CV2897/98 | Sp1#1 wt | 5′- GGGAAGCCACCGGAACCACCCATTTCCTCCCC-3′ |
| CV2899/2900 | Sp1#2 wt | 5’‑CAAGCCGCCCTCATTCGTTGACGACAACCCCTCACCTCAAAAAAAAC-3’ |
| CV2600/01 | Sp1#3 wt | 5’- TCACGCGCCCGCCGCCCTACCTGAGGCCGCCAT-3’ |
| CV2602/03 | Sp1#4 wt | 5’- TCGCGTTCTGCCGCCTCCCGCCTGTGGTGCCTCCT-3’ |
| CV2901/02 | Sp1#5 wt | 5’- CAGATCGAAAGTTCCACCCCTTTCCCTTTCATTC-3’ |
| CV2903/04 | Sp1#6 wt | 5’‑ CTCCGTTGGCTCGGAGCCAGCGACAGCCCATCCTATAGCAC-3’ |
| CV2843/44 | Sp1#1 mut | 5′- GGGAAGCCACCGGAACCA**AA**CATTTCCTCCCC-3′ |
| CV2845/46 | Sp1#2 mut | 5’‑ CAAGCCGCCCTCA**TT**CGTTGACGACAA**AA**CCTCACCTCAAAAAAAAC-3’ |
| CV2671/72 | Sp1#3 mut | 5’- TCACGCGCCCGCCG**AA**CTACCTGAGGCCGCCAT-3’ |
| CV2673/74 | Sp1#4 mut | 5’- TCGCGTTCTGCCGCCT**AA**CGCCTGTGGTGCCTCCT-3’ |
| CV2847/48 | Sp1#5 mut | 5’- CAGATCGAAAGTTCC**TAA**CCTTTCCCTTTCATTC-3’ |
| CV2849/50 | Sp1#6 mut | 5’‑ CTCCGTTGGCTCGGA**TAA**AGCGACA**TAA**CATCCTATAGCAC-3’ |
| CV 1003/1004 | Sp1 consensus wt | 5’-ATTCGATCGGGGCGGGGCGAGC-3’ |
| CV 1004/1005 | Sp1 consensus mut | 5’-ATTCGATCGG**TT**CGGGGCGAGC-3’ |

**Supplementary Table S2. Mutated Sp1 binding sites plasmid constructs.**

| **Primers used in mutagenesis reaction** | **Mutated plasmid constructs obtained** |
| --- | --- |
| CV2843/44 | pGL3-LTR_sense(Sp1#1mut) –luc  pREP-LTR_sense(Sp1#1mut)-luc or LTR_s(Sp1#1mut)1  pREP-LTR_antisense(Sp1#1mut)-luc or LTR_as(Sp1#1mut) |
| CV2845/46 | pGL3-LTR_sense(Sp1#2mut) –luc  pREP-LTR_sense(Sp1#2mut)-luc or LTR_s(Sp1#2mut)  pREP-LTR_antisense(Sp1#2mut)-luc or LTR_as(Sp1#2mut) |
| CV2671/72 | pGL3-LTR_sense(Sp1#3mut) –luc  pREP-LTR_sense(Sp1#3mut)-luc or LTR_s(Sp1#3mut)  pREP-LTR_antisense(Sp1#3mut)-luc or LTR_as(Sp1#3mut) |
| CV2673/74 | pGL3-LTR_sense(Sp1#4mut) –luc  pREP-LTR_sense(Sp1#4mut)-luc or LTR_s(Sp1#4mut)  pREP-LTR_antisense(Sp1#4mut)-luc or LTR_as(Sp1#4mut) |
| CV2847/48 | pGL3-LTR_sense(Sp1#5mut) –luc  pREP-LTR_sense(Sp1#5mut)-luc or LTR_s(Sp1#5mut)  pREP-LTR_antisense(Sp1#5mut)-luc or LTR_as(Sp1#5mut) |
| CV2849/50 | pGL3-LTR_sense(Sp1#6mut) –luc  pREP-LTR_sense(Sp1#6mut)-luc or LTR_s(Sp1#6mut)  pREP-LTR_antisense(Sp1#6mut)-luc or LTR_as(Sp1#6mut) |
| CV2843/44-CV2845/46 | pGL3-LTR_sense(Sp1#1+2mut) –luc  pREP-LTR_sense(Sp1#1+2mut)-luc or LTR_s(Sp1#1+2mut)  pREP-LTR_antisense(Sp1#1+2mut)-luc or LTR_as(Sp1#1+2mut) |
| CV2671/72-CV2673/74 | pGL3-LTR_sense(Sp1#3+4mut) –luc  pREP-LTR_sense(Sp1#3+4mut)-luc or LTR_s(Sp1#3+4mut)  pREP-LTR_antisense(Sp1#3+4mut)-luc or LTR_as(Sp1#3+4mut) |
| CV2847/48-CV2849/50 | pGL3-LTR_sense(Sp1#5+6mut) –luc  pREP-LTR_sense(Sp1#5+6mut)-luc or LTR_s(Sp1#5+6mut)  pREP-LTR_antisense(Sp1#5+6mut)-luc or LTR_as(Sp1#5+6mut) |
| CV2843/44-CV2847/48 | pGL3-LTR_sense(Sp1#1+5mut) –luc  pREP-LTR_sense(Sp1#1+5mut)-luc or LTR_s(Sp1#1+5mut)  pREP-LTR_antisense(Sp1#1+5mut)-luc or LTR_as(Sp1#1+5mut) |
| CV2843/44-CV2845/46-CV2671/72-CV2673/74 | pGL3-LTR_sense(Sp1#1+2+3+4mut) –luc  pREP-LTR_sense(Sp1#1+2+3+4mut)-luc or LTR_s(Sp1#1+2+3+4mut)  pREP-LTR_antisense(Sp1#1+2+3+4mut)-luc or LTR_as(Sp1#1+2+3+4mut) |
| CV2843/44-CV2845/46- CV2847/48-CV2849/50 | pGL3-LTR_sense(Sp1#1+2+5+6mut) –luc  pREP-LTR_sense(Sp1#1+2+5+6mut)-luc or LTR_s(Sp1#1+2+5+6mut)  pREP-LTR_antisense(Sp1#1+2+5+6mut)-luc or LTR_as(Sp1#1+2+5+6mut) |
| CV2843/44-CV2845/46-CV2671/72-CV2673/74-CV2847/48-CV2849/50 | pGL3-LTR_sense(Sp1#tot.mut) –luc  pREP-LTR_sense(Sp1#tot.mut)-luc or LTR_s(Sp1#tot.mut)  pREP-LTR_antisense(Sp1#tot.mut)-luc or LTR_as(Sp1#tot.mut) |

**Supplementary Table S3. Oligonucleotides sequences corresponding to multimerized copies of LTR Sp1 binding sites** (Sp1 site is underlined on the coding strand primer and mutations are highlighted in bold)**.**

| **Oligonucleotide name** | **Sequence of the oligonucleotide** |
| --- | --- |
| Sp1#3wt+4wt | 5’‑ GATATCTCACGCGCCCGCCGCCCTACCTGAGGCCGCCATCCACGCCGGTTGAGTCGCGTTCTGCCGCCTCCCGCCTGTGGTGCCTCCTTCACGCGCCCGCCGCCCTACCTGAGGCCGCCATCCACGCCGGTTGAGTCGCGTTCTGCCGCCTCCCGCCTGTGGTGCCTCCTTCACGCGCCCGCCGCCCTACCTGAGGCCGCCATCCACGCCGGTTGAGTCGCGTTCTGCCGCCTCCCGCCTGTGGTGCCTCCTGATATC-3’ |
| Sp1#3mut+4mut | 5’‑ GATATCTCACGCGCCCGCCG**AA**CTACCTGAGGCCGCCATCCACGCCGGTTGAGTCGCGTTCTGCCGCCT**AA**CGCCTGTGGTGCCTCCTTCACGCGCCCGCCG**AA**CTACCTGAGGCCGCCATCCACGCCGGTTGAGTCGCGTTCTGCCGCCT**AA**CGCCTGTGGTGCCTCCTTCACGCGCCCGCCG**AA**CTACCTGAGGCCGCCATCCACGCCGGTTGAGTCGCGTTCTGCCGCCT**AA**CGCCTGTGGTGCCTCCTGATATC-3’ |
